# Quantitative microbiological risk assessments for *Salmonella* spp. contaminated taiwanese salty chicken in the taiwanese population

**DOI:** 10.1016/j.heliyon.2023.e21467

**Published:** 2023-11-08

**Authors:** Keng-Wen Lien, Meng-Xuan Yang, Min-Pei Ling, Guo-Jane Tsai

**Affiliations:** a Continuing Education Program of Food Biotechnology Applications, National Taitung University, Taitung County, Taiwan; b Department of Food Science, National Taiwan Ocean University, Keelung City, Taiwan

**Keywords:** *Salmonella* spp., Taiwanese salty chicken, Quantitative microbial risk assessment, Probabilistic analysis

## Abstract

The aim of the study is to develop *Salmonella* spp. Quantitative microbial risk assessments (QMRA) and to evaluate the risk of Salmonellosis illness in the Taiwanese population after consumption of Taiwanese salty chicken (TSC). We assume that *Salmonella* spp. May contaminate the fresh raw chicken used in TSC. After transport to the diner, fresh raw chicken is received, cleaned, and surface-washed by diner staff. The TSC is then cooked and sold to consumers. We set four different cross-contamination scenarios to evaluate the contamination level of *Salmonella* spp. In TSC. We used a Monte Carlo simulation method, a probabilistic analysis method, and exceedance risk to evaluate the risk of Salmonellosis illness. When the exceedance risk was 5 %, and taking the Taiwanese population above 19 years old as an example, the rate of contracting Salmonellosis from the consumption of TSC will be 2.94 % (2.94 million per 100 million people) if the chef does not clean their hands, knives, or cutting boards. However, if the chef washes their hands, knives, and cutting boards with cold water and soap, the illness rate of Salmonellosis from consuming TSC will be 1.93E-04 % (193 per 100 million people). Sensitivity analysis indicates that the most important risk factor in the QMRAs of TSC is the temperature of the fresh raw chicken during transportation, following which were the *Salmonella* spp. Residual. If the staff of the diner separates the cooking tools used for raw ingredients and those for cooked food, the illness risk of Salmonellosis will be very low.

## Highlights

• Using quantitative microbial risk assessment method to estimate the potential risks of Taiwanese Salty Chicken to human.
• According to the risk results of *Salmonella* incidence in different scenarios, it can be used as a basis for management.
• Temperature during transportation and cross contamination were the primary risk factors for *Salmonella* contamination.

## 1 Introduction

According to the global epidemiology survey of the World Health Organization (WHO) between 2007 and 2015, the most common causes of food-borne diarrhea cases were Norovirus and *Campylobacter* spp., however, the majority of the cases that resulted in death were due to *Salmonella* spp [1]. In recent years, a large number of *Salmonella* spp.-related critical food-borne cases have occurred internationally. In 2018, the consumption of chicken salad contaminated with *Salmonella enterica* subspecies, *enterica* serovar Typhimurium (*S.* Typhimurium) caused 265 persons to infected and one death in the US; US Centers for Disease Control and Prevention [2] estimates that *Salmonella* causes more foodborne illnesses than any other bacteria. Chicken is a major source of these illnesses. In fact, about 1 in every 25 packages of chicken at the grocery store are contaminated with Salmonella [3].

*Salmonella* spp. Are food pathogen commonly found in poultry products in Taiwan. For example, the food outbreak in 2016 was caused by the cross contamination between raw and roasted duck sold in the market, where *Salmonella* spp*.* Were detected in both the cooked duck and on serving plates. In the same year, *Salmonella* spp. Were detected in 19 bread products sold at a bakery (which were packed with bread, but the main source of salmonella was meat), including fried chicken thigh fillet burgers, smoked chicken burgers, sandwiches, pork floss buns and shallot pork floss rolls (Taiwan Food and Drug Administration [4].

In Taiwan, chicken is commonly contaminated with *Salmonella* spp. And cold chicken dishes may experience cross-contamination between raw and cooked food [4,5]. This is because utensils may not be properly cleaned before and after cooking and no further disinfecting actions are taken after cooking [6]. By using a common Taiwanese food, Taiwanese Salty Chicken (TSC), as an example, this study used quantitative microbial risk assessments (QMRA) to evaluate the *Salmonella* spp. Intake from food in the Taiwanese population, and applied a probabilistic analysis method and an exceedance risk concept to this situation to assess the potential risk of Salmonellosis incidence. This study set the following three aims:

To establish an analysis model for TSC exposed to *Salmonella* spp. To set up hypothetical scenarios and compile literature parameters to estimate the contamination level distribution of *Salmonella* spp. In TSC in scenarios characterized by different hygienic conditions with cross-contamination occurring between raw and cooked food. To establish the probability distribution of *Salmonella* spp. Intake: by combining the contamination level distributions of *Salmonella* spp. In TSC in different scenarios and the consumption of TSC by different gender and age groups within the Taiwanese population, to simulate the probability distribution of *Salmonella* spp. Intake in the Taiwanese population. To assess the potential risk of Salmonellosis incidence in different scenarios: by integrating the probability distribution of *Salmonella* spp. Intake and the dose-response relationship of *Salmonella* spp. To assess the exceedance risk of Salmonellosis incidence in the Taiwanese population when TSC is consumed in different scenarios.

## 2 Materials and methods

### 2.1 Hazard identification

**Fig. 1:**
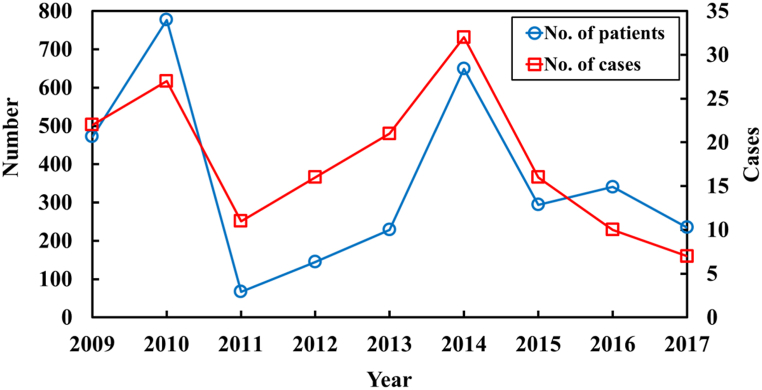
Trend of Salmonella outbreaks in Taiwan over the years. Blue curve is the number of patients in an outbreak and the red curve is the number of Salmonella outbreak cases.

The first step in QMRA is hazard identification. This step involves deciding which microorganisms are of interest in the study and finding out what diseases these microorganisms cause [7]*. Salmonella* spp. Cause a great number of foodborne illnesses every year worldwide. Highly-developed countries are not exceptions, and in Taiwan, *Salmonella* spp. Have even been ranked in the top three causes of bacteria-caused foodborne illnesses over the past few years (TFDA, 2016; 2017; 2018). Gastrointestinal discomfort is the most common manifestation of *Salmonella* outbreaks and most patients do not consult doctors or report its occurrence. As a result, current epidemiology study data may underestimate the actual incidence rate. Trend of Salmonella outbreaks in Taiwan over the years is shown in Fig. 1 [4,5,[8], [9], [10], [11], [12]].

### 2.2 Exposure assessment

**Fig. 2:**
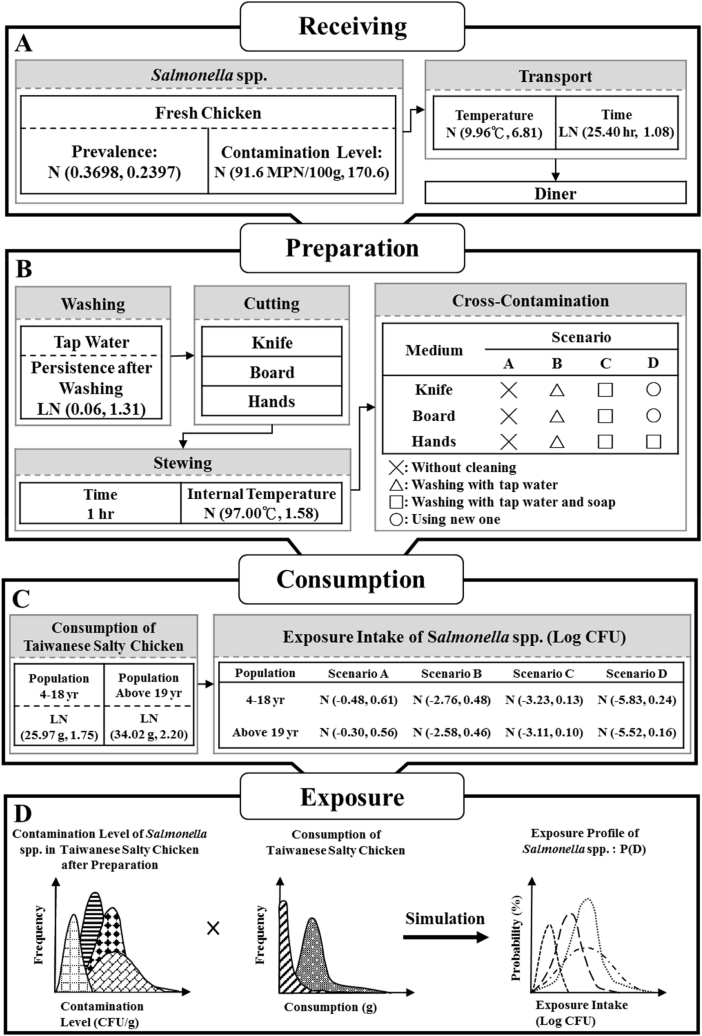
Hypothetical scenarios of *Salmonella* spp. Contamination in Taiwanese Salty Chicken from ingredients to consumption.

In this step, we measure the dose, or the amount of a microorganism individuals are exposed to. This study used fresh chicken contaminated with *Salmonella* spp. As the TSC ingredient, and assumed that the surface of the chicken had been cleaned using drinking water by staff after arriving at the diner following low-temperature delivery, and that cross-contamination between raw and cooked food occurred before cooking. Four cross-contamination scenarios characterized by different hygienic conditions were proposed under such assumptions. By integrating the contamination level of *Salmonella* spp. In TSC and TSC consumption in the Taiwanese population, we simulated and analyzed *Salmonella* spp. Intake in the Taiwanese population. The hypothetical scenarios where TSC were contaminated in various steps from raw ingredients to consumption are shown in Fig. 2.

#### 2.2.1 TSC preparation process

This study assumed that the diner selling salty chicken was a multi-storey building; with the vending department situated on ground level and cooking done on the first level. The cooking utensils were shared within the venue, and staffs were potentially exposed to raw and cooked chicken when moving and operating between floors. To prepare TSC, staff took fresh chicken out from a plastic basket lined with plastic bags containing crushed ice using their bare hands and cleaned the surface of each chicken with drinking water. The chicken was then placed on a chopping board for internal organ removal, including gullet, stomach, intestine and liver, etc. And cleaning of its interior. After that, an incision was made in the neck of the chicken and the whole chicken was dipped into a pot with seasoning and cooked. The seasonings used were taken from a recipe, and included salt, white pepper powder, cooking wine, tangerine peel, star anise, and Chinese cinnamon. Once the cooking was completed, staff would hook the chicken from the opening in the neck and transfer the chicken to the vending department by grabbing the hook and hanging it on a stainless steel bar. The chicken was then cooled and air-dried at room temperature. Upon receiving a customer order, employees took the chicken off the hook, placed it on a chopping board and cut the chicken into pieces with knives; touching the chicken with their bare hands. The chicken pieces were then placed in a container and handed to the consumer.

#### 2.2.2 The initial number of salmonella in specimen, and prevalence of contaminated specimen (contamination level, contamination rate, and bacteria number unit conversion)

*Salmonella* spp. Contamination rate in chicken. In Taiwan, the public generally believes that compared with frozen chicken, TSC prepared with fresh chicken tastes better and is relatively more in agreement with the eating habits of Taiwanese people. However, to fight against the H7N9 prevalence in the Chinese mainland, Taiwan has been pushing to ban the slaughtering of live birds (primarily broiler chickens) in the market since 2013 [13]. Yet in this study used spent laying hens as the ingredient in its hypothetical scenarios and the salty chicken sellers would slaughter the laying hens and process chicken themselves. As a result, this study used the *Salmonella* spp. Contamination data in fresh chicken as the contamination source. Nevertheless, when fresh chicken is stored at room temperature, the temperature favors the growth of *Salmonella*, and the level of contamination of fresh chicken may be higher than that of frozen chicken. Therefore, this study consulted the contamination rate distribution N (0.3698, 0.2397) of *Salmonella* Schwarzengrund in the conventional fresh chicken sold in the market, N stands for normal distribution (Fig. 2A), the mean contamination value is 0.3698 and the standard deviation is 0.2397. This contamination rate assumed to be the initial *Salmonella* spp. Contamination situation of raw chicken [14,15].

Contamination level of *Salmonella* spp. In chicken. According to Taiwanese regulations, the number of *Salmonella* spp. In raw ready-to-eat foods and ready-to-eat foods must be zero [16], and because testing generally gives only qualitative results, there is a detection limit of 1 colony forming unit (CFU)/25 g. As quantitative testing data are missing and it is impossible to identify different serotypes, this study consulted the distribution for contamination level of *Salmonella* spp. N (91.6 Most Probable Number (MPN)/100 g, 170.6) per chicken, in fresh chicken from Beijing, China and assumed that this would be the *Salmonella* spp. Contamination situation in fresh chicken in Taiwan [17].

Bacteria number unit conversion assumption. We consulted the method used in Ref. [18]; where the data from Ref. [19] were analyzed for Goodness-of-Fit using TableCurve 2D® (Version 5.01, AISN Software Inc., Mapleton, OR, USA), and models featuring good rationality and higher coefficients of determination (R^2^) were adopted with priority. The *Salmonella* spp. Contamination level unit was then converted from MPN to CFU using Equation (1):

(Equation 1) y=a(1.0−EXP(−bx))

The model was named Form 1, where x is the contamination level using MPN as the unit, y is the contamination level using CFU as the unit, and parameters a, b and R^2^ are 167.185, 0.006, and 0.880, respectively.

#### 2.2.3 Transportation and washing (temperature and duration of transportation and Salmonella spp. residual rate in chicken after washing)

Temperature and duration of transportation. The temperature and duration of transporting fresh chicken to the diner were determined based on the central temperature distribution (Fig. 2A), N (9.96 °C, 6.81), in the cargo compartment of the refrigerated home delivery vehicles and time distribution, LN (25.40 h, 1.08), during the trip [20]. At this stage, improper temperature control would result in *Salmonella* spp. Propagation, and the quantity of bacteria growth was calculated using Equation (2):

(Equation 2) $$\mathrm{Log}\;N_{1}=\mathrm{Log}N_{0}+\mu \times t/\ln\left(10\right)$$

In this formula, Log N_1_ is the quantity of *Salmonella* spp. Growth (Log CFU), Log N_0_ is the initial *Salmonella* spp. Contamination level (Log CFU), μ is the specific growth rate (h^−1^) and t is time (in hours). The maximum value of the parameter μ in Equation (2) was calculated using Equation (3):

(Equation 3) $$\mu_{\max}=\mu_{\text{opt}}\times \tau \left(\text{pH}\right)\times \rho \left(T\right)$$

In this formula, μ_max_ is the maximum growth rate (h^−1^), μ_opt_ is the optimal growth rate (1.65 h^−1^), τ(pH) is the pH influencing parameter, which has a value of 0.86 (based on pH_min_ = 3.8, pH_opt_ = 7, pH_max_ = 9.5) [21], and ρ(T) is the temperature influencing parameter. The parameter ρ(T) in Equation (3) was calculated using Equation (4):

(Equation 4) $$\rho \left(T\right)=\left[{\left(T-T_{\max})\times (T-T_{\min}\right)}^{2}\right]/\left\{\left(T_{\text{opt}}-T_{\min}\right)\times [\left(T_{\text{opt}}-T_{\min}\right)\times (T-T_{\text{opt}})-\right\}$$

In this formula, ρ(T) is the temperature influencing parameter, T_min_ is the minimum growth temperature (5.2 °C), T_opt_ is the optimal growth temperature (37.0 °C), and T_max_ is the maximum growth temperature (46.2 °C). The growth temperature was assumed to be in the range of 5.2–46.2 °C [22,23].

*Salmonella* spp. Residual rate in chicken after washing. After raw fresh chicken packed with crushed ice was transported to the diner, staff immediately washed the surface of the chickens using tap water to remove dirt, which concurrently removed a portion of the *Salmonella*. This study assumed that the quality of the tap water complied with the standard for Taiwanese drinking water, with the maximum limit of the coliform group being 6 CFU/100 ml and the total bacterial count being 100 CFU/ml. The study also assumed that chicken was not exposed to secondary contamination from pathogens in the tap water and used the *Salmonella* spp. Residual rate distribution (Fig. 2A), LN (0.06, 1.31), as a reference [24,25].

#### *2.2.4* *Preparation* (preparation temperature and time)

The fresh chicken was prepared by dipping the whole bird in a pot. According to the cooking conditions for chicken (Fig. 2B), the distribution of central temperature in the chicken was assumed to be N (97.00 °C, 1.58) and the cooking time was 1 h [26]. Under such conditions, *Salmonella* spp. Levels were reduced due to sterilization at high temperatures, and the D value for the specific temperature at the cooking temperature was calculated using Equation (5):

(Equation 5) $$\mathrm{Log}\;D=\mathrm{Log}D_{0}-\left(T-T_{ref}\right)/Z$$

In this formula, D value is the time (in minutes) of exposure at a given temperature that causes a one-log or 90% reduction in the population of a specific microorganism. Z value is the number of degrees the temperature has to be increased to achieve a tenfold reduction in the D-value. Log D is the Log D value (min) at cooking temperature, Log D_c_ (−0.83 min) is the Log D value when setting 70 °C as the standard temperature, T is the cooking temperature (°C), and z is 9.1 °C. The residual *Salmonella* spp. Levels in chicken after cooking were calculated using Equation (6), after converting the Log D value into the D value:

(Equation 6) $$\mathrm{Log}\;C=\mathrm{Log}C_{0}-t/D$$

In this formula, Log C (Log CFU/g) is the *Salmonella* spp. Level after cooking, Log C_0_ (Log CFU/g) is the *Salmonella* spp. Level in chicken before cooking, t is the cooking time (60 min) and D is the D value at the cooking temperature (min) [27,28]. After the chicken was cooked, the whole cooked TSC was taken out by pulling the hook, and the chicken was then cut into pieces, weighed, and handed to the customer.

#### 2.2.5 Cross-contamination (Salmonella spp. transfer rate through cross-contamination)

*Salmonella* spp. Can be transferred from raw chicken before cooking to the plastic chopping boards, knives, and the hands of the chef; and can then be transferred to the cooked salty chicken as a result of improper cleaning of utensils or hands, or if the utensils were not changed. People taking in these *Salmonella* will contract Salmonellosis. This study considered these three transference pathways, and assumed that the order of the pathways exposed to the raw chicken were plastic chopping boards, the hands of the chef, and knives (Fig. 2B). The study consulted the overall transfer rate data and the relevant parameters are listed in Table 1.

**Table 1:** Summary of the *Salmonella* spp. Intake parameters from consumption of Taiwanese Salty Chicken in the Taiwanese population.

Parameters	Description	Values	Reference
C_0_ (MPN)	*Salmonella* spp. Contamination level of raw chicken	N (91.6 MPN/100 g, 170.6)	[17]
P_0_	*Salmonella* spp. Prevalence of raw chicken	N (0.3698, 0.2397)	[14]; Guo, 2007 [29,30];
t_1_	Time of transportation	LN (25.40 h, 1.08)	[20]
T_1_	Temperature of transportation	N (9.96 °C, 6.81)	[20]
P_W_	Persistence after washing raw chicken	LN (0.06, 1.31)	[25]
t_2_	Time of cooking	60 min	[26]
T_2_	Temperature of cooking	N (97.00 °C, 1.58)	[26]
Log D	Log D-value of *Salmonella* spp. At 70 °C	−0.83 min	[28]
Z	Z-value of *Salmonella* spp. At 70 °C	9.1 °C	[28]
P_RK_	Transfer rate (raw fresh chicken → knife)	10^Normal (0.171,0.162)^%	[31]
P_RB_	Transfer rate (raw fresh chicken → board)	10^Normal (0.171,0.162)^%	[32]
P_RH_	Transfer rate (raw fresh chicken → hands)	10^Normal (0.71,0.42)^%	[33]
P_WK_	Persistance after washing knife with cold water	(10^Normal (−3.51,0.43)^/10^Normal (−3.15,0.49)^)%	[31]
P_WB_	Persistance after washing board with cold water	(10^Normal (−3.51,0.43)^/^10Normal (−3.15,0.49)^)%	van Asselt et al., 2009
P_WH_	Persistance after washing hands with cold water	(10^Normal (−6.46,1.26)^/10^Normal (−2.88,0.68)^)%	van Asselt et al., 2009
P_WSK_	Persistance after washing knife with cold water and soap	(10^Normal (−6.4,1.54)^/10^Normal (−3.15,0.49)^)%	[31]
P_WSB_	Persistance after washing board with cold water and soap	(10^Normal (−6.4,1.54)^/10^Normal (−3.15,0.49)^)%	van Asselt et al., 2009 [31];
P_WSH_	Persistance after washing hands with cold water and soap	(10^Normal (−7.04,0.66)^/10^Normal (−2.88,0.68)^)%	van Asselt et al., 2009 [31];
P_KC_	Transfer rate (knife → Taiwanese salty chicken)	10^Normal (1.458,0.298)^%	[31]
P_BC_	Transfer rate (board → Taiwanese salty chicken)	10^Normal (1.458,0.298)^%	[32]
P_HC_	Transfer rate (hands → Taiwanese salty chicken)	10^Logistic (1.16,0.33)^%	[33]
CR_1_	Consumption rate of Taiwanese salty chicken of Taiwanese population aged 4-18	LN (25.97 g/day, 1.75)	Pan, 2005 [[34], [35], [36]];
CR_2_	Consumption rate of Taiwanese salty chicken of Taiwanese population over 19 years old	LN (34.02 g/day, 2.20)	Pan, 2005 [[34], [35], [36]];

Note: LN = lognormal distribution; N = normal distribution; D-value = decimal reduction time; Z-value = the number of degrees (Celsius or Fahrenheit) required to change a D-value by one factor of ten, MPN = most probable number.

According to the literature, the distribution of the *Salmonella* spp. Transfer rate from fresh raw chicken to knives was 10^Normal (0.171, 0.162)^ % and the distribution of the *Salmonella* spp. Transfer rate from knives to ready-to-eat lettuce, 10^Normal (1.458, 0.298)^ %, was assumed to be the distribution of the transfer rate from knives to TSC. The distribution of the *Salmonella* spp. Residual rate after washing knives with cold water was (10^Normal (−3.51, 0.43)^/10^Normal (−3.15, 0.49)^); and the distribution of the *Salmonella* spp. Residual rate after washing knives with cold water and soap was (10^Normal (−6.4, 1.54)^/10^Normal (−3.15, 0.49)^) [32,33]; van Asselt et al., 2009; [31].

The distribution of the *Salmonella* spp. Transfer rate from fresh raw chicken to plastic chopping boards was 10^Normal (0.171, 0.162)^ % according to the literature; and the distribution of the *Salmonella* spp. Transfer rate from plastic chopping boards to ready-to-eat lettuce, 10^Normal (1.458, 0.298)^ %, was assumed to be the distribution of the transfer rate from plastic chopping boards to TSC. The distribution of the *Salmonella* spp. Residual rate after washing the plastic chopping boards with cold water was (10^Normal (−3.51, 0.43)^/10^Normal (−3.15, 0.49)^); and the distribution of the *Salmonella* spp. Residual rate after washing the plastic chopping boards with cold water and soap was (10^Normal (−6.4, 1.54)^/10^Normal (−3.15, 0.49)^) [32,33]; van Asselt et al., 2009; [31].

The distribution of the *Salmonella* spp. Transfer rate from fresh raw chicken to the hands of the chef was 10^Normal (0.71, 0.42)^ % according to the literature; and the distribution of the *Salmonella* spp. Transfer rate from the hands of the chef to ready-to-eat lettuce, 10^Logistic (1.16, 0.33)^%, was assumed to be the distribution of the transfer rate from the hands of the chef to TSC. The distribution of the *Salmonella* spp. Residual rate after the chef washed their hands with cold water was (10^Normal (−6.46, 1.26)^/10^Normal (−2.88, 0.68)^); the distribution of the *Salmonella* spp. Residual rate after the chef washed their hands with cold water and soap was (10^Normal (−7.04, 0.66)^/10^Normal (−2.88, 0.68)^) [32,33]; van Asselt et al., 2009; [31].

The amount of *Salmonella* spp. In TSC could be calculated from the amount of contaminated with *Salmonella* spp. In the raw chicken, which has gone through the processes of growing, sterilization, and transfer. These formula are provided in the Supplementary Materials.

#### 2.2.6 Consumption (distribution of salty chicken consumption)

This study integrated four years of NAHSIT data; excluding those people who recorded no consumption. We calculated the daily TSC consumption of TSC consumer IDs (Identities) and assumed that it was the TSC consumption per person at each time. The four-year data included data from the National Survey on Changes in Nutrition and Health Status (2005–2008), the Survey on Nutrition and Health Status of Middle School Students in Taiwan (2010), the Survey on Changes in Nutrition and Health Status of High School Students in Taiwan (2011), and the Survey on Changes in Nutrition and Health Status of Primary School Students in Taiwan (2012). For exposed population groups, the study combined the age groups of 4–6 years, 7–12 years, 13–16 years, and 17–18 years into one age group of 4–18 years, and those of 19–65 years and over 65 years into one age group of over 19 years (Fig. 2C), according to the diet section in the national consumption database. As a result, there were a total of two age groups and the distribution of TSC consumption in age groups 4–18 and over 19 were LN (25.97 g/day, 1.75) and LN (34.02 g/day, 2.20), respectively, according to the literature [[34], [35], [36], [37]].

#### 2.2.7 Probabilistic analysis method

By combining the distributions of *Salmonella* spp. Levels and consumption, the distribution of *Salmonella* spp. Intake in the Taiwanese population could be estimated using Equation (7):

Equation 7 $$D_{\text{ij}}=\sum_{j=1}^{n}C_{\text{ij}}\times {\text{CR}}_{\text{ij}}$$

In this formula, D_ij_ is the *Salmonella* spp. Intake (Log CFU) from food (j) by each individual ID (i) from the exposed population groups; C_ij_ is the *Salmonella* spp. Level (Log CFU) in food (j); CR_ij_ is the food (j) consumption (g) of each individual ID (i) or the processing factor (g).

This study used the built-in Monte-Carlo simulation from the statistics software suite Crystal Ball® (Version 5.2.2, Decisioneering, Inc., Denver, CO, USA). Multiple iterative operations were conducted to test the stringency and stability of output values following random sampling, according to which, 10,000 iterative operations could ensure that the values satisfied the stated conditions. Therefore, the iterative operation was set to 10,000. The distribution of *Salmonella* spp. Intake through the consumption of TSC in different age groups in Taiwan was also established (Fig. 2D).

### 2.3 Hazard characterization

According to epidemiology studies, a positive correlation is present between pathogen intake and incidence rate and there is no threshold between the two, meaning even a small amount of pathogen intake causes illness. In this study used a published dose response model in this risk assessment. We consulted the Beta-Poisson Model published by WHO, which consists of a large amount of rational and valid data on the incidence rate of Salmonellosis, including a great number of *Salmonella* spp. Foodborne illnesses outbreak cases from several countries, such as Japan and the US. The model also considered various serotypes and food categories, and established a dose-response relationship between *Salmonella* spp. Level and Salmonellosis incidence rate. Our current study calculated the incidence rate for Salmonellosis based on the Expected Value [38], as shown in Equation (8):

(Equation 8) $$P(R|D)=1-{\left(1+\left(D/\beta \right)\right)}^{-\alpha }$$

In this formula, P (R**|**D) is the incidence rate for Salmonellosis, D is *Salmonella* spp. Intake (CFU), α is 0.1324 (with an upper limit of 0.2274 and lower limit of 0.0763), and β is 51.45 (with an upper limit of 57.96 and lower limit of 38.49).

The primary symptom of concern in this study was acute gastroenteritis. Diagnoses exceeding the scope of this symptom were not considered.

### 2.4 Risk characterization

This study used a probabilistic analysis method and the concept of exceedance risk to assess the potential risk of *Salmonella* spp. Exposure when meat products are consumed by different age groups in Taiwan. This method may correct the exposure and illness scenarios in the study hypothesis and reduce deviations, allowing the risk assessment outcomes to be closer to the real scenario.

### 2.5 Exceedance risk

Exceedance Risk. Using the cumulative probability risk as a basis, the probability of having a higher risk than a certain incidence rate was calculated. A risk curve of cumulative distribution was obtained from the probability risk results, with each dot on the curve representing the exceedance risk of surpassing the hazardous level under that incidence rate. Points on the horizontal axis represent the risk of the incidence of foodborne illness. The vertical axis represents the potential exceedance risk and can be expressed by Equation (9):

Equation 9 $$P\left(E_{D}\right)=P\left(D\right)\times P\left(R|D\right)$$

In this formula, P (E_D_) is the exceedance risk under a certain incidence rate of Salmonellosis; P(D) is the distribution of *Salmonella* spp. Intake from consuming TSC in different age groups in Taiwan; and P (R|D) is the dose-response relationship between *Salmonella* spp. Intake and Salmonellosis incidence rate.

### 2.6 Uncertainty analysis

Due to the lack of complete exposure information in reality, such as the condition of the exposure environment and the differences in each individual's age, health status, and living conditions, as well as the human physiological conditions extrapolated from toxicology data from animal experiments, the use of a single parameter would add many uncertainties to risk assessment, greatly deviating the results from the real situation. Therefore, after obtaining quantitative results for the risk, the uncertainties in these results must be explained in addition, including the hypothesis, integration of qualitative data, and identification of sources.

Uncertainty quantification during risk assessment can be achieved through the Monte-Carlo simulation method and sensitivity analysis. Monte-Carlo simulation sets up the probability distribution of risk assessment and uses random sampling to covert the input uncertainties into forms of probability distribution, such normal distributions, lognormal distributions, and uniform distributions. The uncertainty in risk results can be combined with input probability distributions and can form compound probability distributions, while the uncertainty during risk assessment can be quantified [39].

The sensitivity analysis views parameters influencing risks as independent variables and risks as dependent variables. When fixing all but one parameter, influences from the change of this independent variable on the dependent variables are observed. The aim is to test the sensitivity of output values to changes in variables under a certain model. After sensitivity analysis, suggestions can be provided to improve variables that exert greater influences, so that uncertainties in the output can be reduced drastically and the risk assessment results can be improved [39]. This study used the built-in sensitivity analysis of the statistics software suite Crystal Ball® (Version 5.2.2, Decisioneering, Inc., Denver, CO, USA) and set to exclude negative values to make a random selected, and compared the correlation and contribution percentage of all parameters in the risk assessment. Parameters high in both correlation and contribution percentage had greater influences on the risk assessment outcomes.

## 3 Results

### 3.1 Exposure intake

**Fig. 3:**
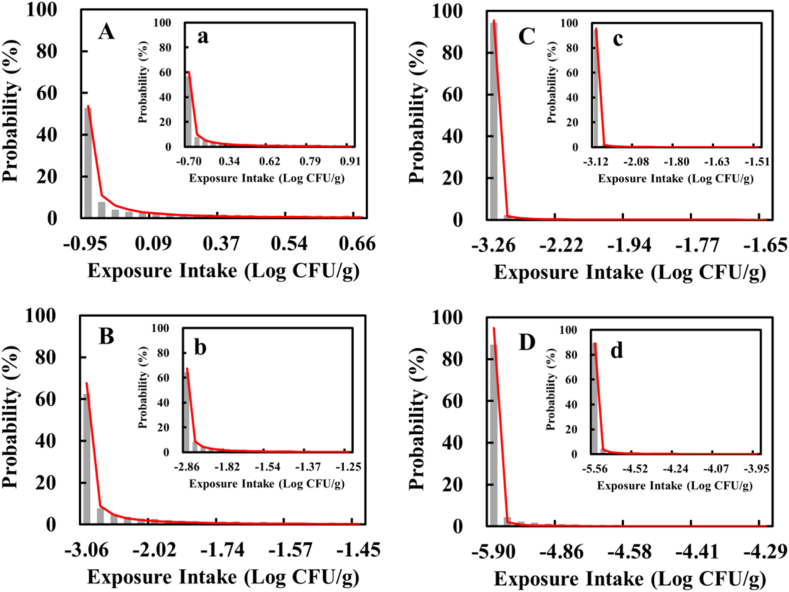
Graphs showing the distribution function of *Salmonella* spp. Intake from consuming Taiwanese Salty Chicken in the age groups of 4–18 years (A–D) and over 19 years (a–d) in Scenarios A, B, C and D (note: in Scenario A, no washing was done for knives, chopping boards, or the chef's hands after cutting raw chicken; in Scenario B, the knives, chopping boards, and the chef's hands were all washed with cold water after cutting raw chicken; in Scenario C, knives, chopping boards, and the chef's hands were all washed with both cold water and soap after cutting raw chicken; and in Scenario D, knives and chopping boards were replaced with new utensils, and the chef's hands were washed with cold water and soap after cutting raw chicken).

The probability distribution of *Salmonella* spp. Intake from consuming TSC in the age group of 4–18 years in the Taiwanese population. For the age group of 4–18 years in the Taiwanese population, according to the probability distribution results of *Salmonella* spp. Intake from TSC: (1) in scenario A (Fig. 3A), where no washing was done for knives, chopping boards, or the chef's hands after cutting raw chicken meat, the average *Salmonella* spp. Intake was −0.48 log CFU per consumption, or 3.3 × 10^5^ CFU *Salmonella* spp. In one million instances of consumption; (2) in scenario B (Fig. 3B), where knives, chopping boards, and the chef's hands were washed with cold water after cutting raw chicken meat, the average *Salmonella* spp. Intake was −2.76 log CFU per consumption, or 1.7 × 10^3^ CFU *Salmonella* spp. In one million instances of consumption; (3) in scenario C (Fig. 3C), where knives, chopping boards, and the chef's hands were washed with both cold water and soap after cutting raw chicken meat, the average *Salmonella* spp. Intake was −3.23 log CFU, or 5.9 × 10^2^ CFU *Salmonella* spp. In one million instances of consumption; and (4) in scenario D (Fig. 3D), where knives and chopping boards were replaced with new utensils and the chef's hands were washed with both cold water and soap after cutting raw chicken meat, the average *Salmonella* spp. Intake was −5.83 log CFU, or 1 CFU *Salmonella* spp. In one million instances of consumption (Fig. 3).

The probability distribution of *Salmonella* spp. Intake from consuming TSC in the group of people over 19 years of age in the Taiwanese population. For the group aged over 19 years in the Taiwanese population, according to the probability distribution results for *Salmonella* spp. Intake from TSC: (1) in scenario A (Fig. 3a), where no washing were done for knives, chopping boards, or the chef's hands after cutting raw chicken meat, the average *Salmonella* spp. Intake was −0.30 Log CFU, or 5.0 × 10^5^ CFU *Salmonella* spp. In one million instances of consumption; (2) in scenario B (Fig. 3b), where knives, chopping boards, and the chef's hands were washed with cold water after cutting raw chicken meat, the average *Salmonella* spp. Intake was −2.58 Log CFU, or 2.6 × 10^3^ CFU *Salmonella* spp. In one million instances of consumption; (3) in scenario C (Fig. 3c), where knives, chopping boards, and the chef's hands were washed with both cold water and soap after cutting raw chicken meat, the average *Salmonella* spp. Intake was 3.11 Log CFU, or 7.8 × 10^2^ CFU *Salmonella* spp. In one million instances of consumption; and (4) in scenario D (Fig. 3d), where knives and chopping boards were replaced with new utensils and the chef's hands were washed with both cold water and soap after cutting raw chicken meat, the average *Salmonella* spp. Intake was −5.52 Log CFU, or 3 CFU *Salmonella* spp. In one million instances of consumption (Fig. 3).

### 3.2 Exceedance risk

#### 3.2.1 The exceedance risk of contracting salmonellosis from consuming TSC in the age group of 4–18 years in taiwanese population

**Fig. 4:**
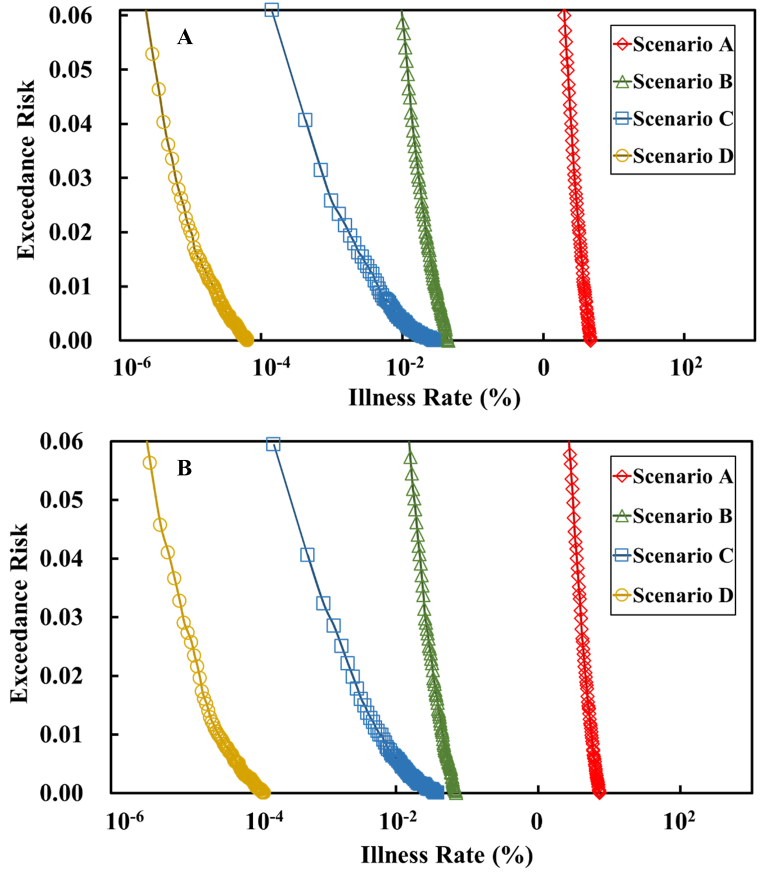
Graphs showing the exceedance risk of contracting Salmonellosis after consuming Taiwanese Salty Chicken in the age groups of 4–18 years (1) and over 19 years (2) in Scenarios A, B, C, and D (note: in Scenario A, no washing was done for knives, chopping boards, or the chef's hands after cutting raw chicken; in Scenario B, knives, chopping boards, and the chef's hands were all washed with cold water after cutting raw chicken; in Scenario C, knives, chopping boards, and the chef's hands were all washed with both cold water and soap after cutting raw chicken; and in Scenario D, knives and chopping boards were replaced with new utensils, and the chef's hands were washed with cold water and soap after cutting raw chicken).

This study use exceedance risk (Equation (9)) for knowing the possibilities that may occur in different specific morbidity scenarios. For instance, assume when consuming a food and had a 99 % chance to ingest 0–1 CFU salmonella, or 1 % chance to ingest over 1 CFU salmonella. And the corresponding incidence rate for an intake of 1 CFU salmonella was 0.034 %, meaning that there was a 99 % chance that the incidence rate would be 0–0.034 %. And a 1 % chance that the incidence rate would exceed 0.034 %. As a result, 1 % is the probability of exceeding a 0.034 % incidence rate of the disease and the exceeding risk under such condition is 1 %. We compared the Salmonellosis incidence rates after consuming TSC in scenarios A, B, C, and D under the exceedance risks of 0.05, 0.03 and 0.01 and the results are shown in Fig. 4.

Under an exceedance risk of 0.05, for the age group of 4–18 years in the Taiwanese population (Fig. 4A): (1) in scenario A, where no washing were done for knives, chopping boards, or the chef's hands after cutting raw chicken meat, the Salmonellosis incidence rate after consuming TSC was 2.14 %, or in other words, the chance of having a Salmonellosis incidence rate higher than 2.13 million affected individuals for every 100 million people was 5 %; (2) in scenario B, where knives, chopping boards, and the chef's hands were washed with cold water after cutting raw chicken meat, the Salmonellosis incidence rate after consuming TSC was 0.0110 %, meaning the chance of having a Salmonellosis incidence rate higher than 11 thousand falling ill for every 100 million people was 5 %; (3) in scenario C, where knives, chopping boards, and the chef's hands were washed with both cold water and soap after cutting raw chicken meat, the Salmonellosis incidence rate after consuming TSC was 0.000142 %, meaning the chance of having a Salmonellosis incidence rate higher than 142 falling ill for every 100 million people was 5 %; and (4) in scenario D, where knives and chopping boards were replaced with new utensils and the chef's hands were washed with both cold water and soap after cutting raw chicken meat, the Salmonellosis incidence rate after consuming TSC was 0.0000029 %, or in other words, the chance of having a Salmonellosis incidence rate higher than three individuals falling ill for every 100 million people was 5 %.

Under an exceedance risk of 0.03, for the age group of 4–18 years in the Taiwanese population (Fig. 4A): (1) the Salmonellosis incidence rate after consuming TSC in scenario A was 2.65 %, or in other words, the chance of having a Salmonellosis incidence rate higher than 2.65 million falling ill for every 100 million people was 3 %; (2) the Salmonellosis incidence rate after consuming TSC in scenario B was 0.0155 %, or in other words, the chance of having a Salmonellosis incidence rate higher than 15 thousand falling ill for every 100 million people was 3 %; (3) the Salmonellosis incidence rate after consuming TSC in scenario C was 0.000708 %, or in other words, the chance of having a Salmonellosis incidence rate higher than 708 falling ill in every 100 million people was 3 %; and (4) the Salmonellosis incidence rate after consuming TSC in scenario D was 0.00000547 %, or in other words, the chance of having a Salmonellosis incidence rate higher than five individuals falling ill for every 100 million people was 3 %.

Under an exceedance risk of 0.01, for the age group of 4–18 years in the Taiwanese population (Fig. 4A): (1) the Salmonellosis incidence rate after consuming TSC in scenario A was 3.50 %, or in other words, the chance of having a Salmonellosis incidence rate higher than 3.49 million falling ill for every 100 million people was 1 %; (2) the Salmonellosis incidence rate after consuming TSC in scenario B was 0.025 %, or in other words, the chance of having a Salmonellosis incidence rate higher than 25 thousand falling ill for every 100 million people was 1 %; (3) the Salmonellosis incidence rate after consuming TSC in scenario C was 0.00297 %, or in other words, the chance of having a Salmonellosis incidence rate higher than 2970 individuals falling ill for every 100 million people was 1 %; and (4) the Salmonellosis incidence rate after consuming TSC in scenario D was 0.0000138 %, or in other words, the chance of having a Salmonellosis incidence rate higher than 14 individuals falling ill for every 100 million people was 1 %.

#### 3.2.2 The exceedance risk of contracting salmonellosis from consuming TSC in the group of individuals over 19 years old in the taiwanese population

This study compared the Salmonellosis incidence rates after consuming TSC in scenarios A, B, C, and D under the exceedance risks of 0.05, 0.03 and 0.01 and the results are shown in Fig. 4. Under an exceedance risk of 0.05, for the group aged over 19 years old in the Taiwanese population (Fig. 4B): (1) the Salmonellosis incidence rate after consuming TSC in scenario A was 2.94 %, or in other words, the chance of having a Salmonellosis incidence rate higher than 2.93 million falling ill for every 100 million people was 5 %; (2) the Salmonellosis incidence rate after consuming TSC in Scenario B was 0.0166 %, or in other words, the chance of having a Salmonellosis incidence rate higher than 16 thousand falling ill for every 100 million people was 5 %; (3) the Salmonellosis incidence rate after consuming TSC in scenario C was 0.000193 %, or in other words, the chance of having a Salmonellosis incidence rate higher than 193 falling ill for every 100 million people was 5 %; and (4) the Salmonellosis incidence rate after consuming TSC in scenario D was 0.00000495 %, or in other words, the chance of having a Salmonellosis incidence rate higher than five individuals falling ill for every 100 million people was 5 %.

Under an exceedance risk of 0.03, for the group aged over 19 years in the Taiwanese population (Fig. 4B): (1) the Salmonellosis incidence rate after consuming TSC in scenario A was 3.78 %, or in other words, the chance of having a Salmonellosis incidence rate higher than 3.78 million falling ill for every 100 million people was 3 %; (2) the Salmonellosis incidence rate after consuming TSC in scenario B was 0.0244 %, or in other words, the chance of having a Salmonellosis incidence rate higher than 24 thousand falling ill for every 100 million people was 3 %; (3) the Salmonellosis incidence rate after consuming TSC in scenario C was 0.000965 %, or in other words, the chance of having a Salmonellosis incidence rate higher than 965 falling ill in every 100 million people was 3 %; and (4) the Salmonellosis incidence rate after consuming TSC in scenario D was 0.00000920 %, or in other words, the chance of having a Salmonellosis incidence rate higher than nine individuals falling ill for every 100 million people was 3 %.

Under an exceedance risk of 0.01, for the group aged over 19 years in the Taiwanese population (Fig. 4B): (1) the Salmonellosis incidence rate after consuming TSC in scenario A was 5.04 %, or in other words, the chance of having a Salmonellosis incidence rate higher than 5.04 million falling ill for every 100 million people was 1 %; (2) the Salmonellosis incidence rate after consuming TSC in scenario B was 0.0392 %, or in other words, the chance of having a Salmonellosis incidence rate higher than 39 thousand falling ill for every 100 million people was 1 %; (3) the Salmonellosis incidence rate after consuming TSC in scenario C was 0.00367 %, or in other words, the chance of having a Salmonellosis incidence rate higher than 3670 individuals falling ill for every 100 million people was 1 %; and (4) the Salmonellosis incidence rate after consuming TSC in scenario D was 0.0000234 %, or in other words, the chance of having a Salmonellosis incidence rate higher than 23 individuals falling ill for every 100 million people was 1 %.

### 3.3 Uncertainty analysis

#### 3.3.1 Scenario uncertainty

Uncertainties resulting from causes including lack of information, too simple scenarios or incomplete hypothetical scenarios may have resulted in overestimated or underestimated risks.

(1) Food preparation and vending venue: this study assumed that the diner was a multi-storey building, with the vending department situated on the ground floor and preparation done on the first. Therefore, the utensils used in the venue were all shared and staff had the chance to touch both raw and cooked chicken when moving and operating between floors. However, in reality, some diners have the vending department clearly separated from the place of food preparation and the distance between the two can be far. In such cases, there might not be utensil sharing or frequent shuttling of things between floors.
(2) Cross contamination in ready-to-use ingredients: this study assumed that each serving of TSC sold contained chicken only and did not consider the issue of cross-contamination between raw and cooked food in the other ingredients, such as shallots, ginger, and chili peppers, which may have resulted in underestimated risks.
(3) Cross contamination from environmental microbes: this study assumed that the cooked chickens were hung over stainless steel bars to be cooled and air dried at room temperature after cooking, and did not consider the microbial issues due to a less clean environment, which may have resulted in underestimated risks.
(4) Rate of *Salmonella* spp. Transfer through cross-contamination: this study only considered three media, namely plastic chopping boards, knives, and the chef's hands and not others, such as wooden chopping boards, ceramic knives, wipe clothes and cash notes. The cleanliness of various media and the order of contact between raw and cooked food with the media could be different in reality.
(5) Physical property parameters: conditions like the pH value of TSC after cooking do not favor bacterial growth, yet this study did not consider the survival rate and growth rate of *Salmonella* after they were transferred onto salty chicken through cross-contamination.
(6) TSC consumption: this study used the consumption data from NAHSIT database and the calculation was made based only on TSC consumers, which means that data with zero consumption of TSC was excluded. Therefore, it was impossible to evaluate the actual consumption situation of the population (whole group). In addition, Taiwanese do not consume TSC every day and TSC don't have the standardized size of servings to refer to. As a result, the consumption could have been overestimated or underestimated. Also, in this study used the amount consumed per day (NAHSIT data), and did not determine the number of servings consumed per year which is not consistent with QMRA best practices.
(7) Use of additives: hydrogen peroxide can be used as a food disinfectant (Food and Drug Administration, 2019). When choosing the ingredients of TSC, chicken parts with darker-looking flesh may be rejected, and some operators may therefore add hydrogen peroxide to offer a better presentation. Nevertheless, the subsequent preparation process of TSC involves cooking at high temperatures without the need of any extra sterilization, and no residual hydrogen peroxide would remain after going through volatilization at high temperatures. This study did not include the use of hydrogen peroxide in the preparation process of the hypothetical scenarios.

#### 3.3.2 Parameter uncertainty

Uncertainties resulting from measurement errors, sampling errors and the use of alternative or hypothetical information, which may have result in overestimated or underestimated risks.

(1) Initial contamination rate of *Salmonella* spp.: this study consulted the contamination rate of *Salmonella* spp. In fresh raw unfrozen chicken reported by Refs. [29,30]; which, instead of multiple *Salmonella* serotypes, only focused on *Salmonella* Schwarzengrund. This might have resulted in underestimated risks.
(2) Initial contamination level of *Salmonella* spp.: this study consulted the contamination level of *Salmonella* spp. In fresh chicken from Beijing, China (Wang et al., 2013), which was not the actual *Salmonella* spp. Quantification results in fresh chicken in Taiwan.
(3) Physical properties of raw chicken: this study calculated the quantity of *Salmonella* spp. Growth using data including maximum growth rate and influencing parameters under optimum environmental conditions such as pH values, while data such as the pH value of raw chicken and water activity were missing.
(4) *Salmonella* spp. Transfer rate through cross contamination: this study assumed that the overall transfer rate was the same as the transfer rates from the literature and did not consider conditions such as the actual exposure frequency and area between the media and raw or cooked food.
(5) Consumption: the NAHSIT consumption database is based on retrospective surveys of food consumption in the previous 24 h and the subjects might have forgotten or entered the wrong information regarding their consumption patterns.

#### 3.3.3 Model uncertainty

The risk models or software models used in this study were only best fit models and did not reflect the real situation and this would cause uncertainties.

(1) Risk model and software: probabilistic analysis method, exceedance risk, and distribution models selected in Table Curve 2D® and Crystal Ball® provided closest estimations based on statistical standards. Yet, there was still a certain level of difference between such estimations and the actual distribution in reality.
(2) Bacterial unit conversion: this study consulted the methods in Ref. [18]; which applied the best fitting method on the research data from McCarthy et al. and converted the units from MPN to CFU. Such conversion does not completely reflect the actual relationships between the two units.
(3) *Salmonella* spp. Growth equation: this study consulted the growth equation in Ref. [23] and calculated the quantity of *Salmonella* spp. Growth after transportation at low temperature. The equation was designed and established according to the optimal growth conditions in the log phase and did not consider the possibilities that the bacteria might be in the lag phase, stationary phase, or death phase. This might have led to overestimation.
(4) Monte-Carlo simulation: this study used the consumption data from the NAHSIT database to establish the TSC consumption distribution in the Taiwanese population. However, this database is limited in subjects and consumption data. A Monte-Carlo simulation was used to estimate the consumption of the whole Taiwanese population based on a small amount of data, whose results might differ from the real situation.
(5) Dose-response relationship: the dose-response relationship referred to by this study was the β-Poisson distribution released by Ref. [38]. This formula was established according to *Salmonella* spp. Food poisoning outbreak cases, where the food samples might have more or less bacteria counted due to improper storage conditions before quantification. This may have caused a large difference between the incidence rate and the actual bacteria intake of the corresponding case, affecting the incidence rate calculation outcomes.

### 3.4 Sensitivity analysis

**Fig. 5:**
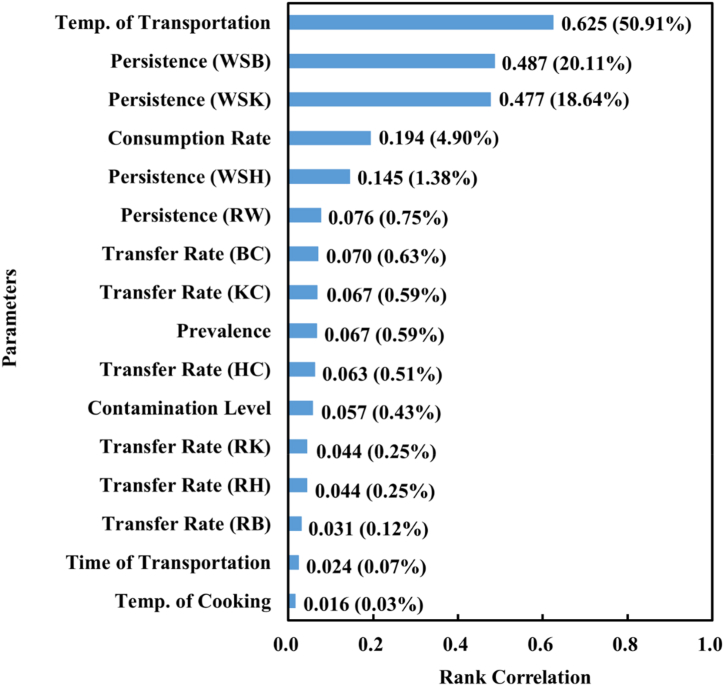
Sensitivity analysis. (Temp. Is temperature, R is raw chicken, C is cooked saltwater chicken, K is knife, B is chopping board, H is chef's hand, W is water washing, WS for cleaning with water and soap).

Taking the TSC consumption in the age group of over 19 years in scenario C as an example (Fig. 5), in this scenario the knives, chopping boards, and the chef's hands were washed with both cold water and soap. The central temperature in the cargo compartment of the refrigerated vehicles showed the highest correlation (0.625, with a contribution percentage of 50.91 %), following which were the *Salmonella* spp. Residual rate after chopping boards were washed with cold water and soap (0.487, with a contribution percentage of 20.11 %), the *Salmonella* spp. Residual rate after washing knives with cold water and soap (0.477, with a contribution percentage of 18.64 %), the TSC consumption in the Taiwanese population (0.194, with a contribution percentage of 4.90 %) and the *Salmonella* spp. Residual rate after the chef washed their hands with cold water and soap (0.145, with a contribution percentage of 1.38 %).

## 4 Discussion

### 4.1 Comparison of Salmonella spp. growth at different temperatures

According to the sensitivity analysis on *Salmonella* spp. Intake from consuming TSC in the group aged over 19 years in the Taiwanese population, the variable showing the highest correlation was the central temperature in the cargo compartment of the refrigerated vehicles, and not the contamination rate or contamination level of *Salmonella* spp. In the fresh raw unfrozen chicken. Improper temperature control during the transportation of this ingredient could result in *Salmonella* spp. Propagation and aggravation of the contamination level. And this parameter (temperature) collected from the distribution of the refrigerated deliveries by Taiwan deliver companies. Through sensitivity analysis, it had shown that food hazards would arise due to improper temperature control during transport and hence industries should work to resolve this issue. As there are no regulations imposed on delivery companies currently, food hazards are present which leads to worries of food safety. Hence the Government should come up with a regulation for manage of cold storage and delivery companies. More, we also compared the differences in the quantity of *Salmonella* spp. Growth by setting different hypothetical temperatures and times.

Hypothetical scenarios: under the hypothesis that the *Salmonella* spp. Contamination rate of fresh raw fresh chicken was 100 % and the contamination level was higher than 1 CFU/g, we set five different temperature scenarios based on routine food storage conditions: room temperature (25 °C), temperature in the fresh food area of a convenience store (18 °C), the lower limit of risk temperature range for food (7 °C), temperature in the refrigerated compartment of a fridge (0 °C), temperature in the frozen compartment of a fridge (below −18 °C) and set the storage time to 12.7 or 25.4 h (according to the literature, this study assumed that the geometric mean of time distribution for the transportation process was 25.4 h, and half of which would be 12.7 h). The quantities of *Salmonella* spp. Growth in these five scenarios were calculated according to Equations (Equation 1), (Equation 2), (Equation 3).

Results of simulation: *Salmonella* spp. Propagation might occur when the temperature conditions of the fresh raw unfrozen chicken in the hypothetical scenarios was within the *Salmonella* spp. Growth temperature range as determined by Equation (3); which would be 5.2–46.2 °C.

Room temperature (25 °C): we assumed that the air-conditioned environmental temperature was 25 °C. When placing fresh raw chicken at 25 °C for 12.7 h, the quantity of *Salmonella* spp. Growth reached about 4.51 Log CFU; when extending the storage time to 25.4 h, the quantity of *Salmonella* spp. Growth reached about 9.02 Log CFU.

Temperature in the fresh food area of a convenience store (18 °C): we assumed that the temperature in the fresh food area of a convenient store was set to 18 °C for the purpose of preserving food freshness and taste. When placing the fresh raw unfrozen chicken at 18 °C for 12.7 h, the quantity of *Salmonella* spp. Growth reached about 2.08 Log CFU; when extending the storage time to 25.4 h, the quantity of *Salmonella* spp. Growth reached about 4.16 Log CFU.

The lower limit of risk temperature range for food (7 °C): the risk temperature range for food is 7–60 °C and most pathogenic microbes can grow and propagate rapidly within this temperature range. When placing fresh raw chicken at 7 °C, the lower temperature limit, for 12.7 h, the quantity of *Salmonella* spp. Growth reached about 1 CFU; when extending the storage time to 25.4 h, the quantity of *Salmonella* spp. Growth remained at about 1 CFU.

Temperature in the refrigerated compartment of a fridge (0 °C): when placing the fresh raw unfrozen chicken at 0 °C in the refrigerated compartment of a fridge for 12.7 h, the quantity of *Salmonella* spp. Residual about 1 CFU; when extending the storage time to 25.4 h, the quantity of *Salmonella* spp. Remained at about 1 CFU.

Temperature in the frozen compartment of a fridge (below −18 °C): when placing the fresh raw unfrozen chicken at −18 °C in the frozen compartment of a fridge for 12.7 h, the quantity of *Salmonella* spp. Residual about 1 CFU; when extending the storage time to 25.4 h, the quantity of *Salmonella* spp. Remained at about 1 CFU.

Summary, according to the comparison on the quantity of *Salmonella* spp. Growth in this section, when the environment temperature was higher than 7 °C, the quantity of *Salmonella* spp. Growth could reach above 4 Log CFU. This result already meets the 50 % bacteria intake condition for contracting Salmonellosis based on the dose-response relationship used by this study. As the number of bacteria multiplies during propagation, longer storage times result in an increased quantity of bacteria growth and the risk of getting the disease also increases; however, with the storage temperature staying below 7 °C, the quantity of *Salmonella* spp. Growth remained close to zero and there would be no need to worry about the aggravation of contamination.

### 4.2 Quantitative microbial risk assessment of Salmonella spp.

The use of QMRA may promote food safety while reducing the incidence rate for food-borne diseases and is helpful for developing domestic and international food trade and establishing pertinent regulation standards. It may also serve as a complementary approach in epidemiology surveys, such as to evaluate missing information and identify the primary cause of an epidemic outbreak. Therefore, each country should conduct its own assessment to improve the model-to-data fitness [38].

Differences of the method used in this study compared to other studies: this study investigated the *Salmonella* spp. Intake from the consumption of TSC sold at diners in the Taiwanese population. The process consisted of steps from the initial contamination present in raw ingredients, transportation of ingredients to diners, washing, preparation, cross-contamination, and consumption. All data were retrieved from literatures and four different hypothetical hygienic conditions were proposed to reflect the cross-contamination scenarios between chopping boards, knives, and the hands of the chef. Consumption data were taken from NAHSIT. A study in China explored the *Salmonella* spp. Intake from consuming home-prepared chicken dishes and the process consisted of steps including the initial contamination present in raw ingredients, transportation of ingredients to consumers’ homes, fridge storage, preparation, cross-contamination, and consumption. This study also sampled chicken from retailer stores that was stored at different temperatures, in different packaging, and sold through different types of markets, and surveyed the chicken preparation situations in the families of university students, such as the time and temperature conditions during the period from purchase to consumption and cross-contamination in their kitchens. The study assumed that cross-contamination was caused by contacts between chopping boards, knives, and the hands of chef used for both raw and cooked chicken or ready-to-eat foods (referring to the foods consumed together with the chicken on the same day). The consumption data were taken from the Chinese Nutrition and Health Survey of 2002 [31]. A study in Canada explored the *Salmonella* spp. Intake from consuming home-prepared chicken breasts, with a process similar to the Chinese study. Using *C*-EnterNet data to establish the distribution model for *Salmonella* spp. Contamination level, the Canadian study calculated the quantity of bacteria growth based on parameters from other studies and assumed that the input time and temperature data were the same as those used for refrigerated food transportation in a Taiwan study [20] and that the preparation temperature was 55–70 °C (there might have been incomplete heating). It also assumed that cross-contamination was caused by contacts between chopping boards and hands used for raw and cooked chicken and conducted conversions on the chicken consumption per person per year in Canada to calculate the fresh chicken breast consumption per person at home [18]. Both our study and the Canadian study converted the *Salmonella* spp. Unit from MPN to CFU for subsequent calculations, and the Salmonellosis incidence rates were both calculated based on the dose-response relationship established according to the β-Poisson model published by Ref. [38].

Differences in *Salmonella* spp. Intake: in the scenario with the least optimal hygienic conditions, scenario A (no washing of chopping boards, knives, or hands), there was at most a 52 % chance to have on average a −0.48 Log CFU *Salmonella* spp. Intake, meaning 11 CFU of *Salmonella* spp. Would be taken in once out of one hundred instances of consumption; while in the scenario with the most ideal hygienic conditions, scenario D (chopping boards and knives were replaced with new utensils and the chef's hands were washed with cold water and soap), there was at most an 86 % chance to have on average a −5.83 Log CFU *Salmonella* spp. Intake, meaning 1 CFU of *Salmonella* spp. Would be taken in once out of one million instances of consumption. According to the Chinese study, in 45.02 % of the subjects' families, purchased raw chicken would be stored at room temperature for 0.5–24 h; 1/3 of the families would use separate chopping boards for raw and cooked food, and 50 % of the remaining 2/3 would use detergent-like soap to wash the chopping boards. The *Salmonella* spp. Contamination rate in chicken dishes was 0.6 %, and the average contamination level in each serving was 0.0310 MPN [31]. The Canadian study did not describe the bacteria intake result [18].

Differences in Salmonellosis incidence rate: for the group aged over 19 years in the Taiwanese population, in the scenario with the least optimal hygienic conditions, scenario A, the Salmonellosis incidence rate from consuming TSC was 2.94 %, meaning there was a 5 % chance for the Salmonellosis incidence rate to be higher than 3 in 100 people falling ill; in the scenario with the best hygienic conditions, scenario D, the incidence rate was 0.00000495 %, meaning there was a 5 % chance for Salmonellosis incidence rate to be higher than 5 in 100 million people falling ill. The results from the Chinese study showed that the average Salmonellosis incidence rate was 0.0120 % from consuming one serving of a chicken dish. However, the distribution of these results was highly skewed, and over 84.2 % consumers had zero risk [31]. According to the results of the Canadian study, the average Salmonellosis incidence rate was 0.0199 % from consuming one serving of chicken breast, and three in every one thousand people would fall ill every year [18].

Uncertainty analysis: the primary factor influencing the risk results in our study was the temperature during the transportation of fresh raw chicken (with a contribution percentage of 50.91 %), followed by the *Salmonella* spp. Residual rate after washing chopping boards (with a contribution percentage of 20.11 %) and *Salmonella* spp. Residual rate after washing knives (with a contribution percentage of 18.64 %). In the Chinese study, 92.6 % of the contamination was due to the chopping boards; if the chef used different chopping boards to process raw and cooked food, the risk could be reduced to 0.00348 %; while if the chopping boards were washed with detergent after processing raw chicken, the risk could be reduced to 0.00574 % [31]. In the Canadian study, the key factors influencing the output results were the cross-contamination coming from chopping boards and hands, the *Salmonella* spp. Contamination level at the retail stage, and preparation with incomplete heating. If the *Salmonella* spp. Level in the raw chicken at the retail stage was reduced by 50 %, Salmonellosis incidence rate could be greatly reduced (about 40 %), and this was followed by cutting down the repeated use of chopping boards by 50 % after processing raw chicken without washing the chopping boards (down about 29 %), and improving the hand washing procedure after processing raw chicken (down about 15 %) [18].

Summary: during the risk assessment processes in different countries, *Salmonella* spp. Contamination levels in raw chicken at the retail stage were all relatively high. This is a parameter that has an important influence on the contamination in the subsequent steps. If the chef modifies their working habits and properly washes chopping boards, knives, and hands to prevent cross-contamination issues from happening, the Salmonellosis incidence rate can be reduced. After comparing our results, collectively speaking, QMRA can be used as a tool for process management and control by organizations to enhance the understanding and knowledge regarding *Salmonella* spp. In personnel involved at the retail and food processing stages.

## 5 Conclusion

If staffs do not properly wash cooking utensils and media in contact with the raw chicken, there will be a relatively high chance of contracting Salmonellosis after consuming TSC. Using the probabilistic analysis method, this study assessed the exceedance risk of contracting Salmonellosis through *Salmonella* spp. Intake from consuming TSC in the age groups of 4–18 years and over 19 years in the Taiwanese population. Using fresh raw unfrozen chicken as the raw ingredient, the study considered three media, including chopping boards, knives, and the chef's hands, and set up four hypothetical cross-contamination scenarios between raw and cooked food. According to the results, with a 5 % probability of occurrence, the incidence rate for the age group of over 19 years in scenario A would be higher than 2.93 million falling ill for every 100 million people; in scenario B, the incidence rate would be higher than 16 thousand falling ill for every 100 million people; in scenario C, the incidence rate would be higher than 193 falling ill for every 100 million people; and in scenario D, the incidence rate would be higher than five people falling ill for every 100 million people. The uncertainty of this exceedance risk would be reduced if one could reduce the central temperature in the cargo compartment of the refrigerated vehicle used for transporting fresh raw chicken and the *Salmonella* spp. Residual rate after washing chopping boards and knives.

Quantitative microbial risk assessment may assist in food safety management. TSC as an example, this study verified that this method could indeed assess the incidence rate of Salmonellosis. Therefore, for quantitative microbial risk assessment, individual models can be established for different food categories and pathogen species in accordance with the specific situation of each country, and the incidence rates of food-borne diseases in different consumer population groups can also be quantified. Our results may serve as complementary data for epidemiology studies or more importantly, provide valid information for policy makers and risk management personnel.

In summary, using quantitative microbial risk assessment, the most critical factors influencing risk results in the model can be identified and strategies can be proposed to effectively reduce the incidence rates of food-borne diseases. Each country should conduct its own quantitative microbial risk assessment in accordance with their people's habits and reality. In the example of TSC, *Salmonella* spp. Intake and Salmonellosis incidence rate were relatively high when consuming food prepared under compromised hygienic conditions, whereas the temperature during transportation and cross contamination were the primary risk influencing factors.

## Funding sources

This work was supported by the 10.13039/100007225Ministry of Science and Technology, Project: 10.13039/501100004663MOST 106-2314-B-019-001.

## CRediT authorship contribution statement

**Keng-Wen Lien:** Conceptualization, Data curation, Methodology, Validation, Writing – original draft, Writing – review & editing, Supervision, Visualization. **Meng-Xuan Yang:** Data curation, Formal analysis, Investigation, Software. **Min-Pei Ling:** Conceptualization, Funding acquisition, Methodology, Project administration, Resources, Software, Supervision, Validation, Visualization, Writing – original draft, Writing – review & editing. **Guo-Jane Tsai:** Methodology, Writing – review & editing, Validation, Visualization.

## Declaration of competing interest

The authors declare that they have no known competing financial interests or personal relationships that could have appeared to influence the work reported in this paper.

## References

[1] World Health Organization, WHO (2015). https://apps.who.int/iris/bitstream/handle/10665/199350/9789241565165_eng.pdf;jsessionid=9CE3D415DDFB37B3589E9AD1E07D1F51?sequence=1.

[2] Centers for Disease Control and Prevention, CDC (2019). https://www.cdc.gov/salmonella/outbreaks-active.html.

[3] Centers for Disease Control and Prevention, CDC (2023). https://www.cdc.gov/foodsafety/communication/salmonella-food.html.

[4] Taiwan Food and Drug Administration, TFDA (2017). http://www.cdway.com.tw/gov/fda/case8/.

[5] Taiwan Food and Drug Administration, TFDA (2014). http://www.cdway.com.tw/gov/fda/case8/.

[6] Chen T.Y. (2021).

[7] World Health Organization (2021). https://www.fao.org/3/cb5006en/cb5006en.pdf.

[8] Taiwan Food and Drug Administration, TFDA (2012). http://www.cdway.com.tw/gov/fda/case8/.

[9] Taiwan Food and Drug Administration, TFDA (2013). http://www.cdway.com.tw/gov/fda/case8/.

[10] Taiwan Food and Drug Administration, TFDA (2015). http://www.cdway.com.tw/gov/fda/case8/.

[11] Taiwan Food and Drug Administration, TFDA (2016). http://www.cdway.com.tw/gov/fda/case8/.

[12] Taiwan Food and Drug Administration, TFDA (2018). http://www.cdway.com.tw/gov/fda/case8/.

[13] Council of Agriculture, Yuan Executive (2019). https://www.coa.gov.tw/ws.php?id=2501081.

[14] Wang C.S. (2003).

[15] Guo J.W. (2007).

[16] Ministry of Health and Welfare (2023). https://law.moj.gov.tw/ENG/LawClass/LawAll.aspx?pcode=L0040142.

[17] Wang Y., Chen Q., Cui S., Xu X., Zhu J., Luo H., Wang D., Li F. (2014). Enumeration and characterization of *Salmonella* isolates from retail chicken carcasses in Beijing, China. Foodborne Pathogens and Disease.

[18] Smadi H., Sargeant J.M. (2013). Quantitative risk assessment of human Salmonellosis in Canadian broiler chicken breast from retail to consumption. Risk Anal..

[19] McCarthy J.A., Thomas H.A., Delaney J.E. (1958). Evaluation of the reliability of coliform density tests. Am. J. Publ. Health.

[20] Kong Y.W. (2012).

[21] Murphy R.Y., Marks B.P., Johnson E.R., Johnson M.G. (1999). Inactivation of Salmonella and Listeria in ground chicken breast meat during thermal processing. J. Food Protect..

[22] Rosso L., Lobry J.R., Bajard S., Flandrois J.P. (1995). Convenient model to describe the combined effects of temperature and pH on microbial growth. Appl. Environ. Microbiol..

[23] Straver J.M., Janssen A.F.W., Linnemann A.R., Van Boekel M.A.J.S., Beumer R.R., Zwietering M.H. (2007). Number of *Salmonella* on chicken breast filet at retail level and its implications for public health risk. J. Food Protect..

[24] Environmental Protection Administration (2017). https://oaout.epa.gov.tw/law/LawContent.aspx?id=FL015512.

[25] Henley S.C., Launchi N., Quinlan J.J. (2018). Survival of *Salmonella* on raw poultry exposed to 10% lemon juice and vinegar washes. Food Control.

[26] Qi J., Liu D.Y., Zhou G.H., Xu X.L. (2017). Characteristic flavor of traditional soup made by stewing Chinese yellow‐feather chickens. J. Food Sci..

[27] Huang L., Juneja V.K. (2001). A new kinetic model for thermal inactivation of microorganisms: development and validation using *Escherichia coli* O157: H7 as a test organism. J. Food Protect..

[28] Van Asselt E.D., Zwietering M.H. (2006). A systematic approach to determine global thermal inactivation parameters for various food pathogens. Int. J. Food Microbiol..

[29] Chen M.H., Wang S.W., Hwang W.Z., Tsai S.J., Hsih Y.C., Chiou C.S., Tsen H.Y. (2010). Contamination of *Salmonella* Schwarzengrund cells in chicken meat from traditional marketplaces in Taiwan and comparison of their antibiograms with those of the human isolates. Poultry Sci..

[30] Chen M.H., Hwang W.Z., Wang S.W., Shih Y.C., Tsen H.Y. (2011). Pulsed field gel electrophoresis (PFGE) analysis for multidrug resistant *Salmonella enterica* serovar Schwarzengrund isolates collected in six years (2000–2005) from retail chicken meat in Taiwan. Food Microbiol..

[31] Zhu J., Bai Y., Wang Y., Song X., Cui S., Xu H., Jiao X., Li F. (2017). A risk assessment of Salmonellosis linked to chicken meals prepared in households of China. Food Control.

[32] Kusumaningrum H.D., Van Asselt E.D., Beumer R.R., Zwietering M.H. (2004). A quantitative analysis of cross-contamination of *Salmonella* and *Campylobacter* spp. via domestic kitchen surfaces. J. Food Protect..

[33] Montville R., Chen Y., Schaffner D.W. (2001). Glove barriers to bacterial cross-contamination between hands to food. J. Food Protect..

[34] Pan W.H. (2010). https://www.hpa.gov.tw/Pages/List.aspx?nodeid=3998.

[35] Pan W.H. (2011). https://www.hpa.gov.tw/Pages/List.aspx?nodeid=3998.

[36] Pan W.H. (2012). https://www.hpa.gov.tw/Pages/List.aspx?nodeid=3998.

[37] Pan W.H. (2008). https://www.hpa.gov.tw/Pages/List.aspx?nodeid=3998.

[38] World Health Organization, WHO (2002). http://www.fao.org/3/a-y4392e.pdf.

[39] Hsu H.T. (2006).

