# Supplementary material for: Quantitative microbiological risk assessments for Salmonella spp. contaminated taiwanese salty chicken in the taiwanese population
Source: Heliyon. 2023 Nov 8;9(11):e21467. doi: 10.1016/j.heliyon.2023.e21467 (PMC10681917; doi:10.1016/j.heliyon.2023.e21467)
Supplement: Multimedia component 1 [file mmc1.docx]

**Supplementary Materials**

**Quantitative Microbiological Risk Assessments for *Salmonella* spp. Contaminated Taiwanese Salty Chicken in the Taiwanese Population**

The amount of *Salmonella* spp. in TSC could be calculated from the amount of contaminated bacteria in the raw chicken, after the process of growth, sterilization, and transfer, etc., and the amount of remaining *Salmonella* spp. in the raw chicken received at the dining room and washed with tap water It can be calculated according to formula 1:

C1 = (C0× R + CT)× PW (Formula 1)

C1 is the amount of *Salmonella* spp. on raw chicken after washing (CFU/g), C0 is the amount of *Salmonella* spp. on raw chicken before washing (CFU/g), R is the contamination rate of *Salmonella* spp. (%), and CT is the growth bacteria of *Salmonella* spp. after home delivery amount (CFU/g), PW is the residual rate of *Salmonella* spp. on raw chicken after washing (%), and PW can be calculated according to formula 2:

PW = (1- PRB) × (1- PRH) × (1- PRK) (Formula 2)

PW is the residual rate of *Salmonella* spp. on the raw chicken after washing (%), PRB is the transfer rate of *Salmonella* spp. from raw chicken to the cutting board (%), PRH is the transfer rate of *Salmonella* spp. from raw chicken to the chef's hands (%), and PRK is *Salmonella* spp. Transfer rate (%) from raw chicken to knife. The amount of *Salmonella* spp. on TSC (cooked meat) after heating and cooking can be calculated according to formula 3:

Log C2 = Log [ (C1×PC) – (t/D) ] (Formula 3)

C2 is the amount of *Salmonella* spp. on TSC (cooked meat) after heating (CFU/g), C1 is the amount of *Salmonella* spp. on raw chicken after washing (CFU/g), PC is the residual rate of *Salmonella* spp. on raw chicken after preliminary cutting (%), t is the cooking time (min), and D is the D value (min) at a specific cooking temperature (°C). The amount of *Salmonella* spp. transferred from the chopping board to the TSC (cooked meat) can be calculated according to formula 4:

CB = C1 × PRB × PWB × PBC (Formula 4)

CB is the amount of *Salmonella* spp. transferred from the cutting board to the TSC (cooked meat) (CFU/g), C1 is the amount of *Salmonella* spp. on the raw chicken after washing (CFU/g), PRB is the transfer rate of *Salmonella* spp. from raw chicken to the cutting board (%), PWB is the residual rate of *Salmonella* spp. after cleaning the cutting board (%), PBC is the transfer rate of *Salmonella* spp. from the cutting board to cooked chicken (%). The amount of *Salmonella* spp. transferred from the chef's hand to the TSC (cooked meat) can be calculated according to formula 5:

CH = C1 × (1- PRB) × PRH × PWH × PHC (Formula 5)

CH is the amount of *Salmonella* spp. (CFU/g) transferred from the chef’s hand to the TSC (cooked meat), C1 is the amount of *Salmonella* spp. on the raw chicken after washing (CFU/g), and PRB is the amount of *Salmonella* spp. transferred from raw chicken to the cutting board. Transfer rate (%), PWB is the residual rate of *Salmonella* spp. after cleaning the cutting board (%), PRH is the transfer rate of *Salmonella* spp. from raw chicken to the chef's hands (%), PWH is the residual rate of *Salmonella* spp. after the chef's hands are washed (%), PHC is the transfer rate (%) of *Salmonella* spp. from chef's hands to TSC (cooked meat). The amount of *Salmonella* spp. transferred from the knife to the TSC (cooked meat) can be calculated according to formula 6:

CK = C1 × (1- PRB) × (1- PRH) × PRK × PWK × PKC (Formula 6)

CK is the amount of *Salmonella* spp. transferred from the knife to the TSC (cooked meat) (CFU/g), C1 is the amount of *Salmonella* spp. on the raw chicken after washing (CFU/g), PRB is the transfer rate of *Salmonella* spp. from raw chicken to the cutting board (%), PWB is the residual rate of *Salmonella* spp. after cleaning the cutting board (%), PRH is the transfer rate of *Salmonella* spp. from raw chicken to the chef's hands (%), PRK is the transfer rate of *Salmonella* spp. from raw chicken to knives (%), PWK is the residual rate (%) of *Salmonella* spp. after cleaning the knife, and PKC is the transfer rate (%) of *Salmonella* spp. from the knife to TSC (cooked meat). Instead, it can be calculated according to Equation 7:

CF = C2 + CB + CH + CK (Formula 7)

CF is the amount of *Salmonella* spp. contaminated in TSC (CFU/g), C2 is the amount of *Salmonella* spp. in TSC (cooked meat) after heating (CFU/g), CB is transferred from the cutting board to TSC (cooked meat) The amount of *Salmonella* spp. (CFU/g), CH is the amount of *Salmonella* spp. transferred from the chef's hand to the TSC (cooked meat) (CFU/g), CK is the amount of *Salmonella* spp. transferred from the knife to the TSC (cooked meat) (CFU/g).
